# Enhancing the Prioritization of Disease-Causing Genes through Tissue Specific Protein Interaction Networks

**DOI:** 10.1371/journal.pcbi.1002690

**Published:** 2012-09-27

**Authors:** Oded Magger, Yedael Y. Waldman, Eytan Ruppin, Roded Sharan

**Affiliations:** 1 Blavatnik School of Computer Science, Tel Aviv University, Tel Aviv, Israel; 2 School of Medicine, Tel Aviv University, Tel Aviv, Israel; Tufts University, United States of America

## Abstract

The prioritization of candidate disease-causing genes is a fundamental challenge in the post-genomic era. Current state of the art methods exploit a protein-protein interaction (PPI) network for this task. They are based on the observation that genes causing phenotypically-similar diseases tend to lie close to one another in a PPI network. However, to date, these methods have used a static picture of human PPIs, while diseases impact specific tissues in which the PPI networks may be dramatically different. Here, for the first time, we perform a large-scale assessment of the contribution of tissue-specific information to gene prioritization. By integrating tissue-specific gene expression data with PPI information, we construct tissue-specific PPI networks for 60 tissues and investigate their prioritization power. We find that tissue-specific PPI networks considerably improve the prioritization results compared to those obtained using a generic PPI network. Furthermore, they allow predicting novel disease-tissue associations, pointing to sub-clinical tissue effects that may escape early detection.

## Author Summary

Identifying the genes causing genetic disease is a key challenge in human health, and a crucial step on the road for developing novel diagnostics and treatments. Modern discovery methods involve genome-wide association studies that reveal regions of the genome where the causal gene is likely to reside, and then prioritizing the candidate genes within these regions and experimentally examining the most promising candidates' potential influence on the disease. Many computational methods were developed to automatically prioritize candidate genes. Some of the most successful methods use a biological network of interacting genes or proteins as an input. However, these networks – and subsequently, these methods – do not take into account the differences between tissues. In other words, a heart disease is analyzed using the same network as a skin disease. We constructed tissue-specific protein interaction networks and explored their effect on an existing prioritization algorithm by comparing the algorithm's performance on the tissue-specific networks and the generic network. We find that integrating tissue-specific data indeed leads to better prioritization. We also used the prioritization results of different tissues in order to suggest new disease-tissue associations.

## Introduction

A fundamental challenge in human health is elucidating the molecular basis of hereditary diseases. Contemporary methods for discovering disease-causing genes usually consist of two steps: first, genome-wide association studies identify genomic intervals that are linked to a disease of interest. Second, the genes within these intervals are examined for their causal relation to the disease [1]–[3]. Experimentally verifying that a gene is associated with a disease is an expensive and time-consuming process, calling for the prioritization of candidate causal genes. A plethora of computational methods were developed to meet this challenge. These methods are often based on system-wide data such as protein interaction networks [4]–[9], gene expression [8], [10]–[12], sequence similarity of genes [13], [14], functional similarity and annotation [8], [12], [13] and more (for a review on these methods see [15], [16]).

Many state of the art algorithms for the gene prioritization problem use protein interaction or functional linkage networks [8], [17], [18], exploiting the tendency of genes causing similar diseases to lie close to one another in the network [16], [19], [20]. However, these methods do not take into account the fact that the vast majority of genetic disorders tend to manifest only in a single or a few tissues [20]–[22]. Typically, the same data sets are used to prioritize genes for a liver disease or a brain disease, even though the molecular landscapes of a Hepatocyte and a neuron are quite dissimilar.

In this work, we incorporate tissue-specific gene expression data into the prioritization process and demonstrate its impact on the prioritization results. The integration is achieved by constructing tissue-specific protein-protein interaction (PPI) networks and employing them in the prioritization. The rationale behind this approach is that many disorders involve a disruption of the ‘molecular fabric’ of different, healthy tissues. From a protein interaction network point of view, this disruption can be often characterized as a perturbation of a gene, corresponding to node removal, or the perturbation of an interaction between two gene products, corresponding to an edge removal [23]. In the context of genetic disorders, even though the underlying harmful mutation exists in all the cells of our body, it most often wreaks havoc only in a few tissues. This tissue selectivity is likely to emerge due to differences in the functionality of the mutated protein within these tissues, its tissue-specific interacting proteins, its abundance and the abundance of its interactors. Thus, the hypothesis underlying the current work is that a tissue specific network, which better represents the actual disease-related tissue, is likely to yield more accurate prioritizations of the diseases it manifests.

The concept of tissue-specific protein interactions is relatively unexplored. Bossi and Lehner [24] analyzed human PPIs in a tissue-specific context, showing that many housekeeping proteins interact with highly tissue-specific proteins, which in turn implies that housekeeping proteins may have tissue-specific roles. Emig and Albrecht [25] expanded this analysis to identify functional differences between tissues, showing that tissue-specific protein interactions are often involved in transmembrane transport and receptor activation. Lin et al [26] analyzed the topological properties of housekeeping and tissue specific proteins within the generic (non tissue-specific) PPI network. Waldman et al. [27] analyzed translation efficiency in humans using PPIs. Using tissue specific PPI networks, they showed that proteins whose genes are translated more efficiently in a specific tissue tend to have more connections within this tissue as compared to other proteins in the same tissue. Lopes et al. [28] created unweighted tissue-specific networks for several separate PPI databases. They used these networks to analyze host-pathogen interactions in a tissue-specific manner. Finally, a proof-of-concept work by Jiang et al. [29] combined five tissue-specific networks taken from Bossi and Lehner [24] to prioritize candidate genes for type 2 Diabetes.

Of note, the lack of tissue specific PPI networks stands in marked difference from the existence of many tissue- and cell-specific variants of other types of biological networks, such as regulatory networks [30]–[32], functional linkage networks [33], [34] and metabolic networks [35]–[37].

The current study is the first large-scale study that aims to enhance the accuracy of existing network-based gene prioritization algorithms by taking into account tissue-specific information. This is achieved by constructing tissue specific PPI networks and utilizing them for gene prioritization instead of the standard, generic PPI network. First, we examine the hypothesis that a gene is likely to be expressed in a healthy tissue for its mutation to clinically manifest in that tissue. Indeed, a large majority (71–83%) of the known disease-causing genes are significantly expressed in the corresponding disease-associated tissue. However, not all disease-associated genes are significantly expressed in the tissues where the disease is manifested. Interestingly, as shown below, we find that most of the remaining genes either have a low expression level across all tissues, or are involved in mediating a response to external stimulus or being involved in multi-cellular developmental processes, and as such are not expected to have high expression under steady-state conditions in the adult tissue.

Focusing on the cases where the disease-related gene is expressed in the associated tissue, we show that integrating tissue specific expression information into a gene prioritization scheme markedly improves its prediction accuracy. Specifically, we generate tissue-specific PPI networks for 60 healthy human tissues using gene expression data from those tissues [38]. We then apply the same candidate prioritization algorithm for both the original and the tissue-specific PPI networks, and compare the performance of each in a cross-validation setting. We find that the tissue-specific variant of the algorithm yields higher area under the receiver-operator characteristics (ROC) curve (AUC) and gives the correct gene a higher ranking than the original variant more often than not. Finally, we extend our method to infer new disease-tissue associations.

## Results

### Tissue-specific expression of disease causing genes

We constructed literature-based gene-disease and disease-tissue association sets. To this end, we retrieved a set of known gene-disease associations from GeneCards [4], [39]. Disease-tissue associations were based on an association matrix generated computationally by Lage et al. [40]. This matrix provides disease-tissue association scores based on the co-occurrence of disease-related and tissue-related terms in PubMed abstracts. These scores are normalized per disease and presented as percentages (See Methods).

For each disease, we assigned the tissue that had the **maximal association score** (**MAS**) with that disease, and filtered diseases whose MAS was below a predefined threshold. For most of the following analyses, we used two thresholds: MAS>8% was the cutoff used by Lage et al., estimated by them to provide 80% assignment accuracy. Filtering by this threshold produced a set of 920 disease-gene associations, spanning 729 diseases and 632 genes. The second threshold, MAS>40%, was estimated by Lage et al to provide ∼90% accuracy. This threshold yielded 349 associations spanning 290 diseases and 269 genes. In both cases, genes whose related disease could not be associated with a specific tissue were removed from the analysis.

Next, we constructed binary tissue-specific gene expression profiles for 60 healthy tissues based on the Novartis Research Foundation Gene Expression Database (GNF) [38] (Methods). Out of 9998 proteins composing the *generic* (not tissue-specific) network, the number of proteins expressed in each tissue varied between 1322 (13%) to 7113 (71%; mean = 4500.8, standard deviation = 1399.3; Supp. Table S1).

**Figure 1:**
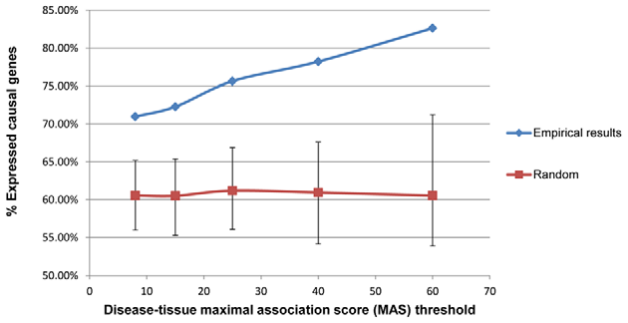
The fraction of disease genes expressed in the disease's assigned tissue correlates with MAS Threshold. The fraction of disease-causing genes expressed in the tissue of their pertaining disease, compared to the random expectation (obtained through a permutation test; Methods), for different MAS thresholds. The error bars represent the minimal and maximal fraction of expressed genes observed at random (over 10,000 permutations) for each MAS threshold. Total number of associations is (from lower to higher MAS): 920, 812, 583, 349 and 167.

For each gene-disease association, we checked whether the causal gene is expressed in the tissue assigned to the disease. Interestingly, we found that a considerable fraction of the causal genes were not expressed in their assigned tissue, ranging between 29% and 17% from MAS>8% to MAS>60%, respectively (Figure 1). Importantly, this fraction is significantly smaller than that expected by chance (38.25% lowly-expressed genes are expected on average across all MAS thresholds, p<1E−5; Methods).

To better understand why disease-causing genes might be lowly expressed in their associated tissues, we studied in detail the 76 lowly-expressed disease-causing genes under a MAS threshold of 40%. First, we analyzed the functional annotations of those genes. Notably, 44 (58%) of the genes were found to be involved in multicellular development processes (GO:0007275, FDR E-value: 1.8E−11), where 36 of those were directly involved in organ development (GO:0048513, FDR E-value: 7.1E−12). Hence, mutations in these genes might disrupt their early embryonic activity leading to pathologies in adult tissues regardless of their expression in these mature tissues. In addition, 17 (22%) of the genes were involved in cellular response to stimulus (GO: 0051716, FDR E-value: 1.8E−4) and, therefore, may not be expressed under normal conditions.

We also found that disease-causing genes that were lowly expressed in the tissue associated to the disease tended to be expressed in fewer tissues than expected (12.1 tissues on average compared to 17.5 at random, p<1E−5; Methods). In addition, these genes exhibited lower mRNA levels across all tissues than the expected levels (150.4 versus 224.8 Affymetrix average difference (AD) units expected by chance, p<1E−5, see Methods). We believe that these observations may partly explain the phenomenon of low-expression of genes in the pertaining disease tissues, as further elaborated upon in the Discussion section. Henceforth, we focused on the majority of disease-causing genes where the gene is indeed expressed in its associated tissue (denoted ‘the expressed disease genes association set’).

### Constructing tissue-specific protein-protein interaction networks

**Figure 2:**
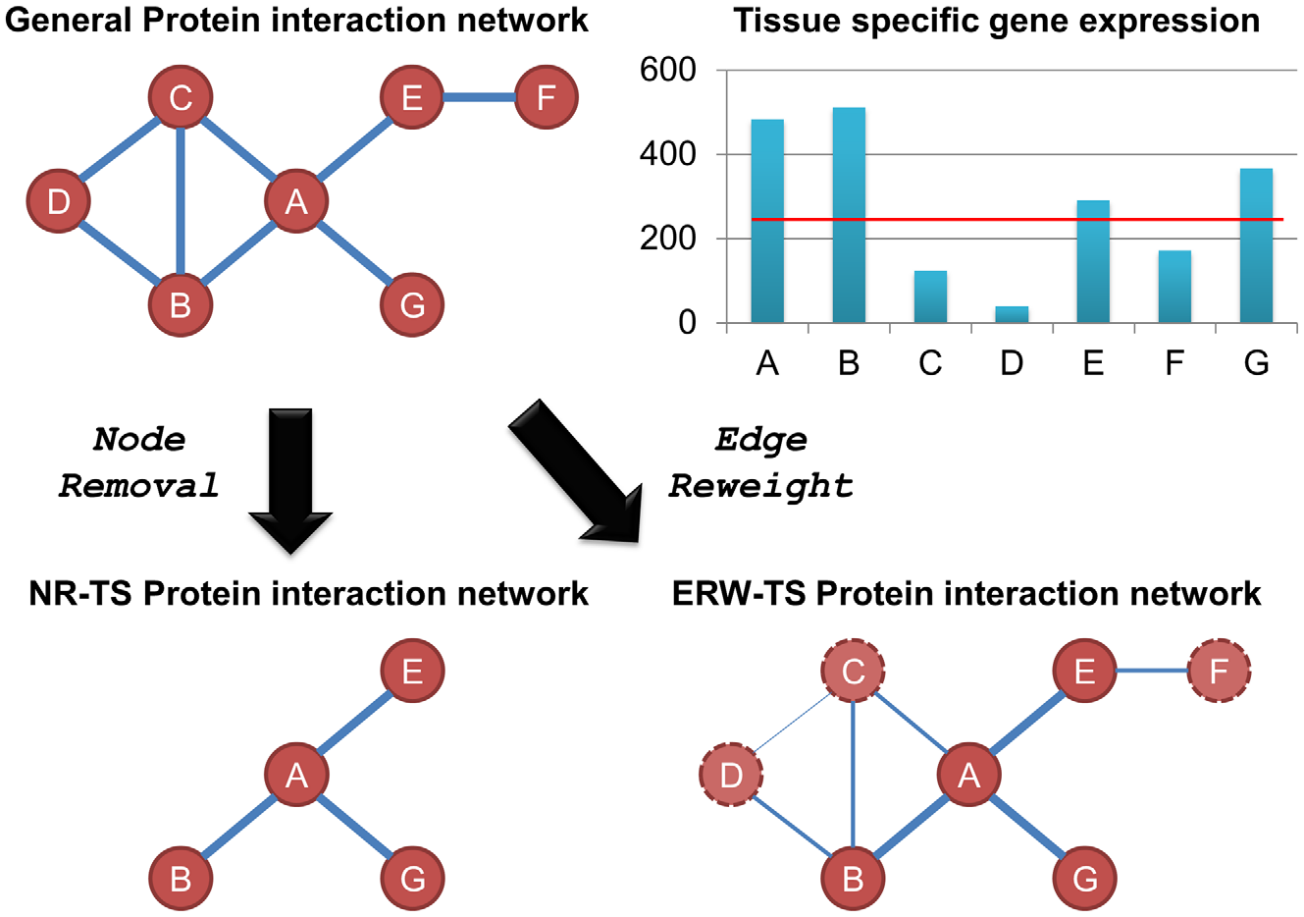
A summary of tissue-specific PPI network reconstruction methods. First, we determine the set of expressed genes in a given tissue based on an expression cutoff of 200 Affymetrix AD units. The set of expressed genes is then superimposed on the general PPI using one of two strategies: (a) Node Removal – removing genes which are considered unexpressed from the network. (b) Edge Reweight - Reducing the weight of an edge connecting one or two unexpressed genes. This results in a tissue specific PPI network.

We considered two methods for converting the generic PPI network into a tissue-specific network using a given tissue-specific expression profile. These methods are summarized in Figure 2 and discussed below.

A naïve method, titled “**Node Removal**” (**NR**), was used previously by Bossi and Lehner [24], Waldman et al [27] and Lopes et al [28]. By this method, a tissue-specific PPI network is generated by removing from the network proteins that are not expressed in the relevant tissue. Notably, such a scheme dramatically changes the connectivity of the network: while a generic PPI networks tends to have a single giant connected component containing most of the network's nodes [41], the NR network is shattered into a relatively small giant component and many small connected components. While the generic human network is composed of 91 connected components with the giant component covering 98% of the network (9796 proteins), the resulting tissue-specific networks have 545 connected components on average, with the average size of the giant component being 3907 proteins. The other components are very small – a few surpass 4 proteins, and none surpass 10.

The number of interactions also drops, from 41049 in the generic network to 14257.21 on average (Range: 2026[4.9%]–27571[67.1%], standard deviation: 6195.4). The amount of expressed proteins and retained interactions in the network have a strong positive correlation (Pearson: p = 0.9939). Moreover, there's also a similarly strong positive correlation between the amount of expressed proteins and average interactions per expressed protein at the tissue (Pearson: p = 0.9803), suggesting that the power-law distribution of interactions is retained. See Supp. Table S1 for the detailed properties of the tissue-specific Node Removal networks.

The second tissue-specific network reconstruction method, novel to this work, is titled ‘**Edge Reweight**’ (**ERW**). By this method, we do not alter the topology of the generic network, but rather modify the edge weights to reflect the probability that the corresponding interactions take place at the specific tissue. In brief, the original confidence score of an edge is multiplied by a penalty factor, *rw*, for each interacting protein that is not expressed in the tissue (see Methods for full details). Note that when *rw* = 0, the ERW network becomes the NR network; conversely, when *rw* = 1, the ERW network is identical to the original PPI network. Thus, varying values of *rw* allow us to control just how tissue specific the network is.

The NR and ERW (with *rw* = 0.1) PPI networks are publicly available as supporting material (Datasets S1, S2, S3).

### Predicting causal genes using tissue-specific Protein interaction networks

In order to prioritize candidate disease genes, we used the PRINCE prioritization algorithm, which we have previously shown to compare favorably to other state-of-the-art algorithms [4], [17]. For completeness, we include a brief description of PRINCE below; for a detailed description see [4].

PRINCE receives a weighted PPI network, a disease-disease phenotypic similarity network and a disease-gene association set as inputs. Given a query disease, PRINCE assigns a prior score to genes associated with known diseases that are phenotypically similar to the query. This score is then propagated through a PPI network in an iterative process, culminating in a smooth scoring function where the score of a node tends to be similar to the scores of its neighboring nodes.

In more detail, let ***q*** be the query disease and denote by ***F(v)*** the prioritization score to be computed for gene v. Let ***Y(v)*** be the prior score for gene *v* (with respect to *q*), defined as 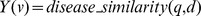 if *v* is associated with a disease *d*, and 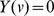 if no disease is associated with *v*. If *v* is known to be associated with multiple diseases, then disease that is most similar to *q* is chosen.

*F(v)* is calculated as a linear combination of *Y(v)* and the scores of *v*'s neighboring nodes:
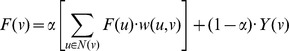
Where *N(v)* is the set of nodes adjacent to *v* in the network, *w(u,v)* is the confidence of the interaction between u and v, and 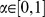 is a parameter controlling the relative importance of the network vs. the prior information.

We applied PRINCE to score disease-causing genes using both the original PPI network and the tissue-specific networks built with the **NR** and **ERW** strategies, and used a leave-one-out cross validation to assess the performance of PRINCE given each network as input, in terms of AUC (Methods). For ERW, we used the MAS>40% association set as a benchmark to identify the optimal *rw* parameter, by constructing multiple TS-ERW (Tissue-Specific Edge ReWeight) networks with varying values of *rw* and comparing their AUCs (Figure S1 and Methods). By Figure S1, the performance has a single peak situated at the lower end of *rw's range*: ***rw*** **= 0.1** for the entire disease-gene association set, and ***rw*** **= 0.001** for the expressed disease genes association set. In the following results presentation we will concentrate on these two choices of the *rw* parameter, as well as on *rw* = 0.5 which is situated in mid-range and thus represents a moderately tissue-specific network. As explained above, we focused the performance evaluation on the subset of disease-gene associations where the causal genes are known to be expressed in the associated tissue (see Figure S3 and Text S1 for an analysis over the entire association set).

**Figure 3:**
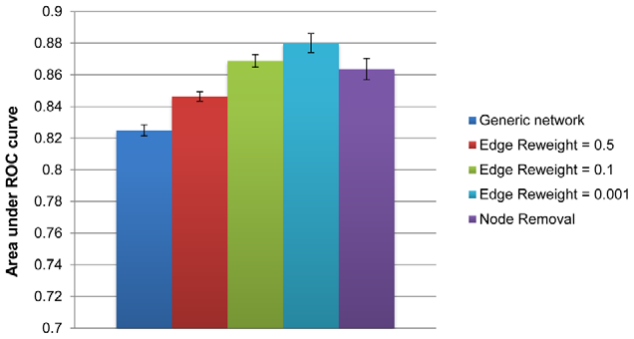
Comparing generic and tissue-specific PPIs' performance in candidate disease genes prioritization. Performance comparison between the generic and different variants of tissue-specific PRINCE, according to the ROC Area under curve (AUC) of causal gene prediction in a leave-one-out cross validation test. Error bars represent the standard deviation of AUC values obtained when replacing leave-one-out with 25-fold cross validation of ten random partitions. Results are for a disease-tissue MAS threshold of 40%.

For MAS>40%, the AUC of the original, generic PPI network (0.825) was lower than that of each representative tissue-specific network (0.85–0.88). The results, summarized in Figure 3 and Figure S2, point to a moderate yet considerable improvement. Among the tissue-specific networks, **TS-ERW** with ***rw*** **= 0.5**, which is the most similar to the original network, exhibits the smallest improvement. The improvement peaks for **TS-ERW** with ***rw*** **= 0.001**. **TS-NR** (tissue-specific node removal) and **TS-ERW** with ***rw*** **= 0.1** networks have comparable AUC values.

We further inspected the cross-validation results by comparing the ranking of true causal genes in the generic network to the tissue-specific networks on a case-to-case basis, in order to estimate how often the tissue-specific data improves the prioritization. Instead of bundling all of the cross-validation results together, we regarded every test case (disease-gene association) in the data set separately, and compared the ranking given to the actual causal gene by PRINCE using the generic and the tissue-specific PPI networks. We found that for every tested MAS threshold, both ERW and NR tissue-specific PRINCE ranked true associations higher than the generic PRINCE in a majority of the cases (Table 1). This also holds true when considering the entire association set, with the exception of NR at MAS>8% (Supp. Table S2). For example, when choosing a MAS threshold of 8% (the same threshold used in [40]) and reweight parameter *rw* = 0.1, we observe that TS-ERW PRINCE gives better ranks to 288 (47%) true associations, whereas the generic network PRINCE gives better ranks to only 58 (9.5%) true associations. 266 associations are identically ranked under both network types. To assess the significance of the improved rankings, we performed a Wilcoxon signed-rank test between the rankings of true causal genes provided by generic PRINCE and every tissue-specific variant. As evident from Table 1, the rankings obtained by the tissue-specific variants significantly outperform the ranking of the generic variant (p<1E−8). Similar trends were observed when analyzing the entire association set (Table S2).

**Table 1:** Evaluation of generic and tissue-specific gene prioritization methods according to their ranking of the true causal genes.

		#cases of better ranking			
MAS threshold	Tissue-specific network type	Tissue-specific	Tie	Generic	Wilcoxon signed-rank test p-value
**8%**	NR	295	203	103	2.09e−15
**8%**	ERW, *rw* = 0.001	291	233	88	9.12e−26
**8%**	ERW, *rw* = 0.1	288	266	58	1.88e−37
**8%**	ERW, *rw* = 0.5	248	334	30	8.85e−37
**40%**	NR	125	91	40	7.68e−9
**40%**	ERW, *rw* = 0.001	124	102	30	1.24e−14
**40%**	ERW, *rw* = 0.1	122	117	17	2.84e−17
**40%**	ERW, *rw* = 0.5	103	145	8	6.34e−17

The table presents a case-to-case comparison of the ranking provided by generic and tissue-specific PRINCE, as well as the statistical significance of this comparison using Wilcoxon signed-rank test.

### Inferring disease-tissue associations

Having the ability to predict the effects of disease genes on specific tissues, naturally gives rise to the question: given a disease (a collection of disease-causing genes), what tissues are most likely to be affected? This is of particular interest, since while the overt clinical manifestations of a disease are usually well-known, in many cases it may have more subtle, sub-clinical tissue effects that may escape early detection. Such alterations may manifest themselves at later stages of the disease, and may be wrongly attributed to other potential complications and confounding factors, instead of the original disease, which can serve at least as an important predisposing factor.

To investigate this potential scenario in depth, we developed a method to computationally infer disease-tissue associations using the framework presented in the previous section. For a given query disease, we applied TS-ERW PRINCE (*rw* = 0.1, chosen for its robustly positive results for both disease-gene association sets presented in the paper) once for every tissue, using the tissue's modified PPI network as input. We then ranked the tissues according to the **relative rank** PRINCE assigns to the causal gene in the tissues' respective networks (for results obtained using the **absolute score** PRINCE assigns to the causal gene see Supp. Text S1 and Figure S4). For example, given a disease *d* and a known causal gene *g*, if g is ranked 4^th^. when using PRINCE with the Kidney PPI and 6^th^. when using PRINCE with the Heart PPI, then the kidney is considered more strongly associated with *d* than the heart.

**Figure 4:**
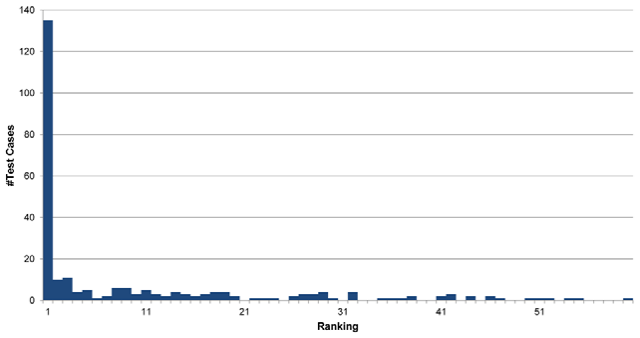
Evaluation of tissue-disease association prediction. The histogram shows the distribution of our disease-tissue ranking for the tissues assigned by Lage et al. in every test case (disease-gene association). As can be seen, in more than half of the cases the associated tissue was predicted first among all other tissues.

We compared our predicted disease-tissue associations to the data collected by Lage et al. For every disease with MAS>40%, we checked what ranking was given to the tissue which was assigned the highest association score by Lage et al (Figure 4). In 53% of the cases the tissue predicted by Lage was ranked first by us as well (p<0.013, see Methods). These results further show the power of tissue specific PPI approach to detect tissue specific disease involvement. Obviously, such analyses could not have been performed using the generic PRINCE method, which is oblivious of the tissue-specific information.

## Discussion

In the current study we aimed to infer disease causing genes using tissue-specific PPI networks. Most previous studies that used these networks to infer causal genes were based on generic PPI networks and ignored differences between tissues [19]. Nevertheless, this generic approach may be limited as there are significant differences in expression patterns between tissues, both with respect to mRNA as well as in protein levels [38], [42]. These differences imply that different tissues have different active PPI networks: a specific interaction may take place in some tissues while not in others, based on the expression distribution of the interacting proteins [24]. Moreover, these differences may explain why, in many cases, a disease may affect a specific tissue and not others: the same protein may be active in specific tissues and inactive in others, or can have different function in different tissues based on its different neighbors in the different networks. Following these observations, we decided to examine the utility of building and incorporating tissue specific PPI networks in our analysis. Adding tissue specificity information for various diseases [40] we were able to perform a tissue specific inference of disease causing genes.

We used the PRINCE algorithm for gene prioritization and contrasted between generic and tissue specific PPIs. We found that the tissue specific approach enhances the performance of the algorithm. In our analysis we used two different methods for tissue specific PPI networks construction that yield different gene prioritization performance. We observed that better results were obtained when modifying the weights of the networks edges (using the ERW method) compared to following the more drastic approach of removing lowly-expressed proteins from the network (using the NR method). There may be several explanations for these differences. First, it may be related to PRINCE algorithm. A global network-based algorithm such as PRINCE is expected to be less successful when applied to a more disconnected network, such as those generated by the NR approach. Moreover, even for other algorithms that are based on local inference which is not propagated, ERW may be proven more appropriate. NR is a very strict method, eliminating every unexpressed protein, while ERW assigns a continuous value for the interaction based on the expression of the two interacting proteins. Thus, the former is likely to be less robust to noisy data such as gene expression [43]–[45]).

One might suggest that there is no need to generate tissue specific PPI networks for tissue specific prioritization. Rather, one might use the generic PRINCE and then, in a post-processing manner, assign the lowest possible ranks to the lowly-expressed genes in the tissue being investigated. While such an attenuation approach performs poorly when applied to the entire gene-disease association set (AUC = 0.755, Figure S3, ‘Unexpressed genes attenuated’), it improves over the standard PRINCE when its application is restricted to associations that involve genes that are expressed in the relevant tissue. As shown in Figure S5, for MAS>40% this approach yields an AUC of 0.897, albeit significantly smaller than the attenuated version of TS-ERW (AUC = 0.905, Wilcoxon sign rank P-value = 0.02 for *rw* = 0.1).

Interestingly, as a preprocessing step for the tissue specific PRINCE algorithm, we found that a considerable fraction of disease genes are not expressed in the tissue associated with the disease. There may be several explanations for this observation. First, it may reflect an error in measurements, either of the expression microarray or the computational inference of disease-tissue association. Nevertheless, such a substantial fraction of genes is more likely to reflect a true biological observation. For example, a protein may be active although having lower mRNA levels. Posttranscriptional modifications or higher translational efficiency may also result in higher protein levels or longer protein half-lives [46], [47]. In addition to putative differences between mRNA and protein levels, obviously, there may be proteins who perform their function in relatively low levels Indeed, we found that many of the genes unexpressed at their disease's assigned tissue also have low overall expression levels, suggesting that these genes might still be expressed at functional level in the diseased tissue.

Another possibility may be that the damage to the tissue was caused by a disruption of the protein function within the tissue in earlier developmental stages. Supporting this hypothesis we found that lowly-expressed disease causing genes are enriched with developmental annotations such as multicellular development processes (GO:0007275) and organ development (GO:0048513), and with stimulus response annotations (GO:0051716). Hence, the protein may not be active in the adult tissues (as manifested by its expression pattern), but a mutation in the genes may alter normal development of the tissue or may prevent the normal response of the tissue to stress or other stimuli, resulting in a disease. Finally, due to the complexity and the dependencies between tissues in a multi-tissue organism, a mutation in a protein active in one tissue may result in clinical pathology in another tissue. For example, Vitamin D – dependent rickets 1A (MIM: 264700) is primarily a bone disorder, but it is caused by a mutation in the gene *CYP27B1*, which is active in the kidney and participates in the hydroxylation of Vitamin D into its active form, Calcitriol [48]. Overall, the role of lowly expressed genes in causing disease in a given tissue is a rather complex one and deserves a separate analysis that is beyond the current scope.

Some limitations of the current analysis should be mentioned. First, a direct tissue specific measure of protein abundance would be more adequate than mRNA levels as a measure for the presence and hence the activation and functionality of a protein in a tissue. However, despite the best efforts of the scientific community, compendiums of human tissue-specific protein abundance levels across multiple tissues are not nearly as comprehensive as the mRNA expression dataset we use, both in tissue scope, gene coverage and quantitative resolution [42]. In addition, the mutual expression of two possibly interacting proteins does not guarantee that the interaction will take place, and there are other factors that also should be considered such as, most prominently, the proteins' phosphorylation state. Nevertheless, even given these limitations, our tissue specific approach performs better than the generic approach. As large scale data on tissue specific protein abundance and cellular localization will become available, it will be interesting to repeat the analysis reported here to see whether it yields better predictions, as may be expected.

In recent years, PPI networks were shown to be a powerful tool in many fields of molecular biology, such as predicting protein annotation and more [19], [49], [50]. We hope that the results of this study will encourage future studies to utilize tissue-specific PPI networks to further increase their predictive and explanatory power.

## Methods

### Tissue specific gene expression

We downloaded the Novartis Research Foundation Gene Expression Database (GNF) tissue-specific gene expression data set [38] from the Gene Expression Omnibus (http://www.ncbi.nlm.nih.gov/geo/) (GDS596). We averaged replicas and for each gene took the probe with the maximal expression level. We used 60 non-diseased tissues for which disease-tissue association data existed (out of 79 tissues). Following [24], [51], a gene was considered expressed in a tissue if its expression in that tissue was at least 200 Affymetrix average-difference (AD) units. Similarly, a protein was defined to be present in a tissue if the corresponding gene was expressed there.

### Disease-tissue associations

The disease-tissue association matrix was contributed by Kasper Lage [40]. Lage et al. estimated the association of a tissue and a disease by measuring their co-occurrences in PubMed abstracts. Specifically, the association score was computed using Ochiai's coefficient (OC) [52], and then normalized by the sum of all OCs for the same disease. Subsequently, each disease was associated with the tissue that attained the highest association score. A computationally-generated disease-tissue association set was used since at this time there was no large-scale, manually curated disease-tissue association set available.

### Analysis of lowly expressed causal genes

For 76 disease genes that were lowly-expressed or not expressed (i.e., expression below 200 AD units) in the tissue associated with the relevant disease, we conducted functional enrichment analysis using the DAVID web server [53], [54]. To test if these genes tend to be lowly expressed across all tissues, we generated a random set of genes that are lowly-expressed on the same disease tissues as the original set. I.e., for each original disease-causing gene that is lowly expressed in the associated tissue, we randomly selected another gene that is also lowly-expressed in that tissue. Next, we computed for each gene the number of tissues in which it was expressed (expression breadth) and compared the resulting distribution to that of a random set, repeating the comparison across 10,000 random sets built similarly. In the same manner we also evaluated the significance of the distribution of average expression level of the lowly-expressed genes.

To estimate the number of disease genes that are expected to be lowly-expressed at an assigned tissue at random, we computed this quantity for 10,000 permutations of the tissue assignment vector taken for a given MAS threshold. We permuted the vector instead of picking a tissue from a uniform distribution for every disease, in order to maintain the bias of tissues that tend to be associated with many diseases (e.g. skin diseases, cardiac diseases). Since the fraction measured experimentally was lower than those resulting from the 10,000 permutations, the estimated p-value is p<1E−5.

### Generic and tissue–specific network construction

We constructed a weighted human PPI network with 9,998 proteins and 41,702 interactions. The network is based on three high throughput experiments [55]–[57] and the HPRD database [58]. The interactions were assigned confidence scores based on the experimental evidence available for each interaction using a logistic regression model adapted from [59]. We considered two ways of obtaining tissue-specific networks: **Node-removal tissue-specific PPI network** was derived by removing from the original PPI network proteins that are not expressed in the relevant tissue, and all of the edges adjacent to them. The remaining edges were retained, along with their weights. In an **edge – reweight tissue-specific PPI network**, the confidence of each interaction represents the probability that the interaction takes place within a given tissue.

We now describe in detail the reweighting scheme that we used. Our underlying assumption was that an interaction between proteins P_1_ and P_2_ occurs at a specific tissue t if only if P_1_ and P_2_ interact in the general network and are both expressed at tissue t. Denote the event that proteins P_i_ and P_j_ interact in the generic network as I_i,j_, and the event that protein P_i_ is expressed in tissue t as X(i,t). Now, a gene is considered expressed in a given tissue if its measured expression level in that tissue is above 200 AD units. However, expression data is often noisy [45], [51] so there is a chance that a gene not passing this cutoff is still expressed (we assume that if a gene passes the threshold then it is indeed expressed in the given tissue). If we denote this probability by *rw*, then
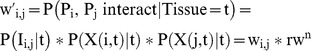
where w_ij_ is the original weight of the interaction and n is the number (0–2) of lowly-expressed genes in tissue t out of {P_i_,P_j_}. Thus, conversion of the generic PPI weight to a tissue specific PPI weight using the edge reweight method involves multiplying an edge's weight by *rw* if one of its adjacent genes is not expressed in the tissue, and by 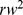 if neither of the edge's adjacent genes are expressed in the tissue.
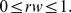



### Prioritization and performance evaluation

We extracted from GeneCards [39] 1347 gene-disease associations. 938 of these associations included diseases for which tissue association information was available. This narrowed gene-disease association set spans 744 distinct genetic disorders and 637 distinct causal genes. For most experiments, diseases with a maximal tissue association score (MAS) below a certain threshold were filtered out. Disease information was taken from the Online Mendelian Inheritance in Man (OMIM) knowledgebase [60]. The disease similarity network was constructed and pre-processed as described in [4]. PPI edge weights were also normalized by the degree of their adjacent protein. The algorithm parameters were the same as in [4]: α = 0.9, c = −15 and 10 iterations.

To evaluate the performance of the different variants of PRINCE, we used a leave-one-out cross validation procedure. In each cross-validation trial, a single disease-gene association, <*g,d*> was removed from the association set. In addition, any other disease-gene association involving *g* was removed to avoid the trivial case where mutations in the same gene cause two very similar disorders. PRINCE was then executed to score the nodes of the network. For the purpose of performance assessment, we constructed an artificial genomic interval of 100 genes which are part of the generic network and are located around g on the genomic sequence, for every *g*. The scores assigned to these 100 genes were compared to *g*'s score. Note that for NR networks, unexpressed genes may still appear in the artificial interval, but they automatically gain a score of ‘0’. Using an artificial linkage interval enabled us to simulate the real-life scenario where prioritization is done only on genes residing within the genomic interval association with a disease.

To generate the ROC curve, we bundled together all of the scores from all of the cross validation trials, sorting them from highest to lowest and recording true- and false- positive rates at various score cutoffs. The actual causal genes were considered positive, and the rest of the genes were considered negative.

For case-to-case rank comparison, we considered each trial separately, and counted in how many trials did the tissue-specific PRINCE gave the actual causal gene a better rank compared to the entire network PRINCE, in how many times tissue-specific PRINCE gave a worse rank, and in how many cases both input networks yielded the same rank (Table 1).

To assess the significance of the difference between the different AUCs, we employed 25-fold cross validation. We performed random partitions and used the standard deviation as error bars in Figure 3a. The statistical significance of the ranking differences was evaluated via Wilcoxon signed-rank tests (Tables 1 and S2).

To fine-tune the *rw* parameter, we constructed sets of TS-ERW networks for varying values of *rw* and repeated the leave-one-out cross validation procedure for each set, for both the entire disease-gene association set and the expressed disease genes association set. We filtered the diseases-gene association sets with a MAS>40% disease-tissue association threshold. We sampled the value of *rw* at constant intervals in the range [0,1]. Having observed that the AUC peaks at the lowest non-zero value of *rw* (0.1), we proceeded to sample smaller values of *rw*, each one smaller than the previous in an order of magnitude. We stopped this procedure when we observed a decline in performance at both sets and convergence to the AUC yielded by *rw = 0*.

### Inferring disease-tissue associations

We filtered the disease-gene association set with a MAS>40% disease-tissue association threshold. The 40% threshold was picked in order to retain only high-confidence associations (∼90% estimated accuracy). We considered only disease-tissue associations where the causal gene is known to be expressed in the tissue assigned by Lage et al. [40].

For each disease-gene pair, we removed the association and ran PRINCE with the same definitions and parameters as the previous section. We repeated the procedure once per tissue, using that tissue's TS-ERW PPI with *rw* = 0.1 (A value shown to produce stable positive results for both association sets) as an input for PRINCE. We then assessed PRINCE's performance for every tissue using the **relative rank** PRINCE assigns the causal gene. Finally, we sorted the tissues' according to the PRINCE rank and ranked them accordingly.

We evaluated the correlation of our tissue ranking with the tissues given the highest association score by Lage et al. for each disease (denoted ‘*assigned tissue*’ from now on). For every disease-gene association, we checked the ranking we gave to the assigned tissue.

To provide an estimated p-value for the high number of highly-ranked assigned tissues, we performed a permutation test as follows: For every disease-gene association, we assigned at random a tissue to the disease, selecting from the tissues where the causal gene is expressed (to counter the bias caused from focusing on disease-gene associations where the gene is expressed in the assigned tissue), and marked the ranking we give the randomly assigned tissue'. When using the ‘ranking by PRINCE rank’ scheme, we counted how many times the random tissue was ranked first. We repeated this procedure 1000 times.

## Supporting Information

Dataset S1 **Entrez ids of network genes.** At Datasets S2, S3, the network genes are indexed from 1 to 9998. This file contains the Entrez ids of these genes, sorted by their index.
(XLSX)

Dataset S2 **The Edge-Reweight tissue-specific PPI network.** The file is divided to 60 sections. Section headers are denoted with ‘#’. Each row represents a single interaction. The first and second columns are the interacting genes' indices, and the third column is the interaction confidence, after reweight. Confidences are not normalized. This network was generated with *rw* = 0.1.
(ZIP)

Dataset S3 **The Node-Removal tissue-specific PPI network.** The file is divided to 60 sections. Section headers are denoted with ‘#’. Each row represents a single interaction. The first and second columns are the interacting genes' indices, and the third column is the interaction confidence. Confidences are not normalized. Note that even though some of the genes are removed at each network, the gene indices are the same as in the other Datasets.
(ZIP)

Figure S1 **Benchmarking the rw parameter.** Comparing the ROC AUC obtained by a leave-one-out cross validation trials for varying values of *rw*, using (A) The expressed disease-genes association set and (B) the entire disease-gene association set. Disease-tissue associations were filtered using a MAS>40% threshold.
(PNG)

Figure S2 **ROC curve comparison of generic and tissue-specific variants of PRINCE.** These ROC curves yielded the ROC AUC values presented in Figure 3. The curves are the output of a leave-one-out cross validation test, using the expressed disease-genes association set and filtering disease-tissue associations with a MAS threshold of 40%.
(PNG)

Figure S3 **Comparing generic and tissue-specific PPIs' performance in disease genes prioritization using the entire disease-gene association set.** Performance comparison between generic and different variants of tissue-specific PRINCE according to ROC Area Under Curve of causal gene prediction in a leave-one-out cross validation test, using the entire disease-gene association data set. The comparison also includes a special variant of generic PRINCE where genes unexpressed at the tissue get an automatic score of 0 (Orange column, described at the third paragraph of the discussion section). Test cases where disease-tissue association had a MAS lower than 40% were discarded.
(PNG)

Figure S4 **Evaluation of tissue-disease association inference using the Absolute Score scheme.** The histogram shows the distribution of our disease-tissue ranking for the tissues assigned by Lage et al, when we use the Absolute Score ranking scheme instead of the Relative Rank ranking scheme. In this scheme, tissues are ordered according to the score PRINCE assigns to the actual causal gene at every tissue. As can be seen, this scheme leads to a more fine-grained differentiation of tissue ranking.
(PNG)

Figure S5 **Comparing generic and tissue-specific PPIs' performance using post-process attenuation of unexpressed genes.** A performance comparison between the generic and different variants of tissue-specific PRINCE, using a special version of PRINCE where, in a post-processing step, the scores of all genes not expressed in the relevant tissue is set to 0. These AUC values were obtained by a leave-one-out cross validation trial using the expressed disease-genes set and a MAS threshold of 40%.
(PNG)

Table S1 **Topological properties of the tissue-specific Node Removal networks.**
(XLSX)

Table S2 **Evaluation of generic and tissue-specific gene prioritization methods using the entire disease-gene association set.** The table presents a case-to-case comparison of the ranking provided by generic and tissue-specific PRINCE, as well as the statistical significance of this comparison using Wilcoxon signed-rank test.
(PDF)

Text S1 **Supporting results.** Describing the analysis of causal genes prioritizations with tissue-specific networks using the entire disease-gene association set, as well as disease-tissue association inference using the Absolute Score tissue ranking scheme.
(PDF)
