# Supplementary figures and images for: Enhancing the Prioritization of Disease-Causing Genes through Tissue Specific Protein Interaction Networks
Source: PLoS Comput Biol. 2012 Sep 27;8(9):e1002690. doi: 10.1371/journal.pcbi.1002690 (PMC3459874; doi:10.1371/journal.pcbi.1002690)

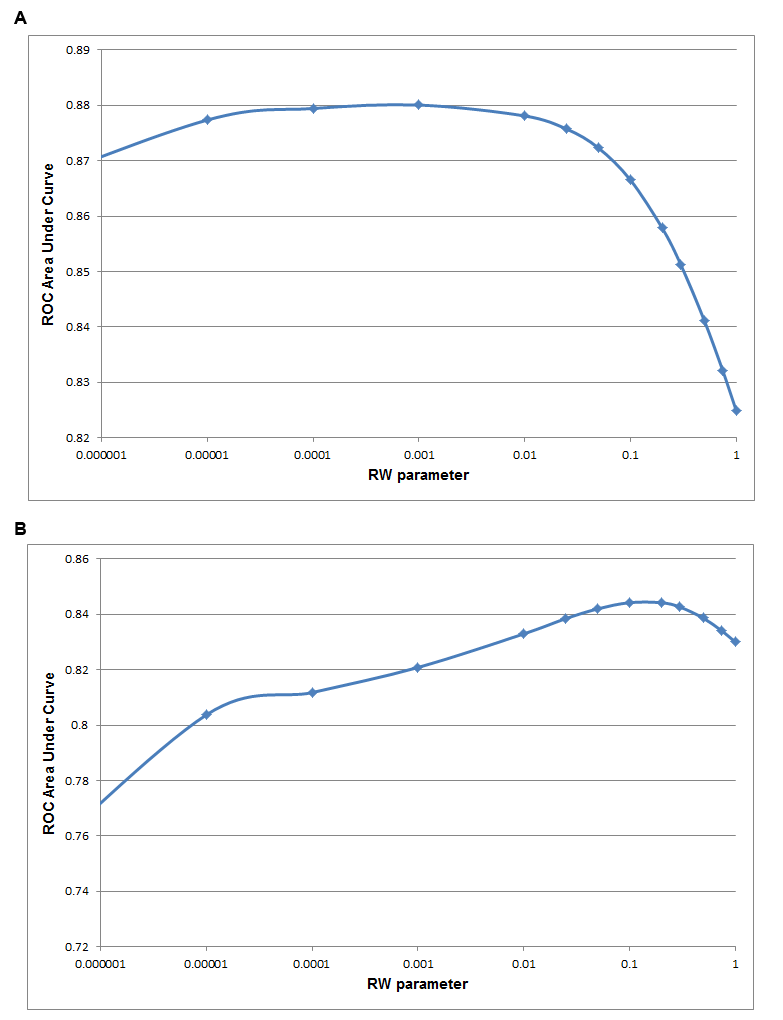

Supplement: Figure S1 — Benchmarking the rw parameter. Comparing the ROC AUC obtained by a leave-one-out cross validation trials for varying values of rw, using (A) The expressed disease-genes association set and (B) the entire disease-gene association set. Disease-tissue associations were filtered using a MAS>40% threshold. (PNG) [file pcbi.1002690.s004.png]

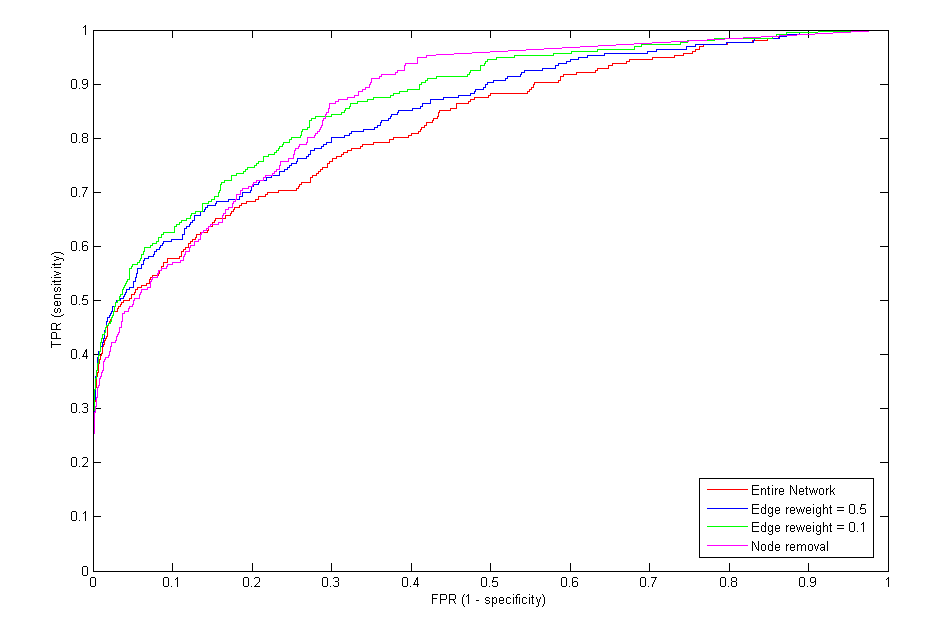

Supplement: Figure S2 — ROC curve comparison of generic and tissue-specific variants of PRINCE. These ROC curves yielded the ROC AUC values presented in Figure 3. The curves are the output of a leave-one-out cross validation test, using the expressed disease-genes association set and filtering disease-tissue associations with a MAS threshold of 40%. (PNG) [file pcbi.1002690.s005.png]

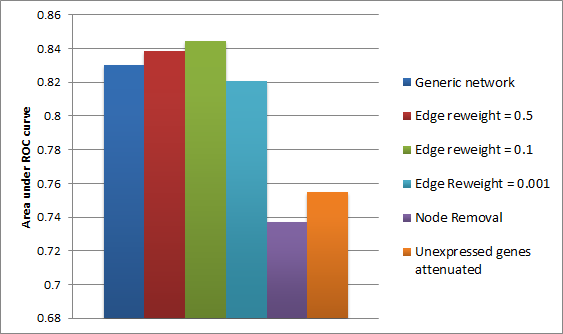

Supplement: Figure S3 — Comparing generic and tissue-specific PPIs' performance in disease genes prioritization using the entire disease-gene association set. Performance comparison between generic and different variants of tissue-specific PRINCE according to ROC Area Under Curve of causal gene prediction in a leave-one-out cross validation test, using the entire disease-gene association data set. The comparison also includes a special variant of generic PRINCE where genes unexpressed at the tissue get an automatic score of 0 (Orange column, described at the third paragraph of the discussion section). Test cases where disease-tissue association had a MAS lower than 40% were discarded. (PNG) [file pcbi.1002690.s006.png]

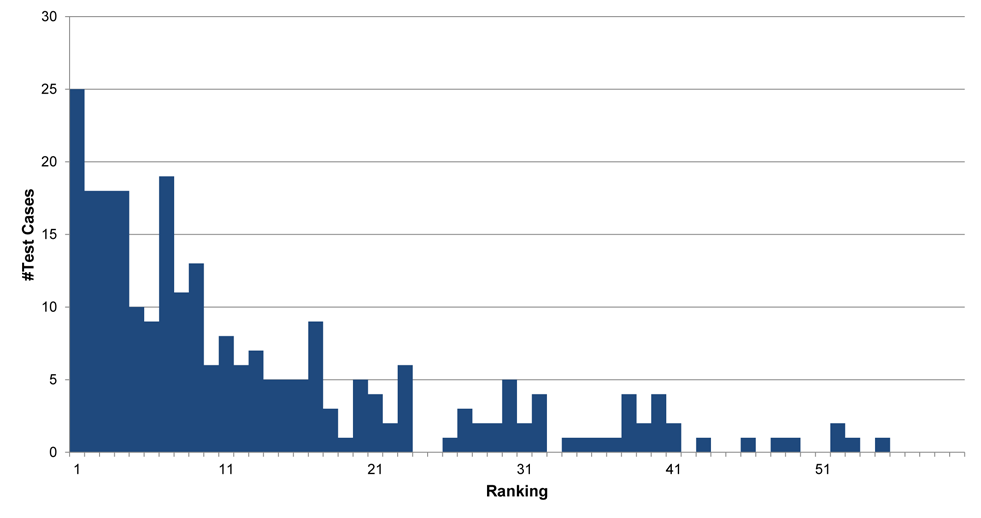

Supplement: Figure S4 — Evaluation of tissue-disease association inference using the Absolute Score scheme. The histogram shows the distribution of our disease-tissue ranking for the tissues assigned by Lage et al, when we use the Absolute Score ranking scheme instead of the Relative Rank ranking scheme. In this scheme, tissues are ordered according to the score PRINCE assigns to the actual causal gene at every tissue. As can be seen, this scheme leads to a more fine-grained differentiation of tissue ranking. (PNG) [file pcbi.1002690.s007.png]

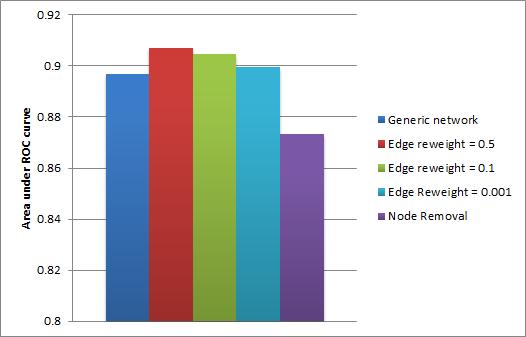

Supplement: Figure S5 — Comparing generic and tissue-specific PPIs' performance using post-process attenuation of unexpressed genes. A performance comparison between the generic and different variants of tissue-specific PRINCE, using a special version of PRINCE where, in a post-processing step, the scores of all genes not expressed in the relevant tissue is set to 0. These AUC values were obtained by a leave-one-out cross validation trial using the expressed disease-genes set and a MAS threshold of 40%. (PNG) [file pcbi.1002690.s008.png]
