# Supplementary material for: Enhancing the Prioritization of Disease-Causing Genes through Tissue Specific Protein Interaction Networks
Source: PLoS Comput Biol. 2012 Sep 27;8(9):e1002690. doi: 10.1371/journal.pcbi.1002690 (PMC3459874; doi:10.1371/journal.pcbi.1002690)
Supplement: Table S2 — Evaluation of generic and tissue-specific gene prioritization methods using the entire disease-gene association set. The table presents a case-to-case comparison of the ranking provided by generic and tissue-specific PRINCE, as well as the statistical significance of this comparison using Wilcoxon signed-rank test. (PDF) [file pcbi.1002690.s010.pdf]

MAS threshold	Tissue-specific network type	#cases of better ranking			Wilcoxon signed-rank test p-value
		**Tissue-specific**	**Tie**	**Generic**	
8%	NR	312	204	363	7.6e-17
8%	ERW, *rw* = 0.001	305	273	301	0.0036
8%	ERW, *rw* = 0.1	298	357	224	0.01
8%	ERW, *rw* = 0.5	261	465	153	7.54e-7
40%	NR	129	93	110	0.004
40%	ERW, *rw* = 0.001	127	114	91	0.5466
40%	ERW, *rw* = 0.1	141	126	65	3.48e-4
40%	ERW, *rw* = 0.5	107	183	42	6.56e-7
