# Supplementary material for: Enhancing the Prioritization of Disease-Causing Genes through Tissue Specific Protein Interaction Networks
Source: PLoS Comput Biol. 2012 Sep 27;8(9):e1002690. doi: 10.1371/journal.pcbi.1002690 (PMC3459874; doi:10.1371/journal.pcbi.1002690)
Supplement: Text S1 — Supporting results. Describing the analysis of causal genes prioritizations with tissue-specific networks using the entire disease-gene association set, as well as disease-tissue association inference using the Absolute Score tissue ranking scheme. (PDF) [file pcbi.1002690.s011.pdf]

SUPPORTING TEXT

Predicting causal genes using tissue-specific Protein interaction networks for the entire association set

We repeated the cross-validation test as described in the results section, this time over the entire disease-gene association set including diseases where the true causal gene is not expressed in the disease-affected tissue (Figure S1).

We observed that the area under curve (AUC) of the **ERW-TS** networks is still higher than the AUC of the original network, but by a less decisive margin (0.845 vs. 0.83). The best performance of an ERW-TS network was observed for a parameter of *rw* = 0.1, which is higher (thus implying less tissue-specific network) than the parameter yielding the best results for the subset of associations where the causal gene is expressed in the assigned tissue. The **NR-TS** networks yielded a low AUC curve due to the fact that unexpressed causal genes were automatically assigned a score of 0 by PRINCE.

We also repeated the case-by-case rank comparison analysis and the Wilcoxon signed-rank test (Supp. Table S2). Here, except for the case of NR-TS and MAS = 8%, the tissue-specific methods rank the causal gene higher more often than not, with a statistically significant divergence of ranking distribution. We observed that the margin becomes smaller for lower MAS thresholds, which is likely due to the linear relationship between MAS and the fraction of expressed causal genes (Figure 1).

Disease-Tissue ranking – alternative tissue ranking scheme.

In addition to ranking tissues in relation to a given disease by PRINCE relative rank, we also tried ranking the tissues according to the **absolute score** PRINCE assigns to the causal gene, e.g. if a gene gets a score of 0.5 when using the heart network and 0.8 when using the kidney network, then kidney will be ranked higher than heart.

This method gives a more fine-grained separation of tissues and the results seem less decisive accordingly (Figure S4). Nonetheless, at a considerable portion of the diseases, the tissue associated according to Lage et al was given a high ranking: in 10% of the cases the assigned tissue was ranked first (p = 0.01), and in 38% of the cases, the tissue was among the top 6 tissues (top 10%; p = 0.002). Estimating the p-value was done using permutation test as described in the main text’s Methods section.
